# Sex Differences Among Patients Undergoing Transcatheter Tricuspid Valve Repair Using the Edge-to-Edge Technique

**DOI:** 10.3390/jcdd11110372

**Published:** 2024-11-19

**Authors:** Mhd Nawar Alachkar, Astrid Eichelsdörfer, Hesham Mady, Andrea Milzi, Rakan Saadoun, Lukas Krygier, Steffen Schnupp, Christian Mahnkopf

**Affiliations:** 1 Department of Cardiology and Angiology, Klinikum Coburg, Ketschendorfer Str. 33, 96450 Coburg, Germany; astrid.eichelsdoerfer@regiomed-kliniken.de (A.E.); hesham.mady@regiomed-kliniken.de (H.M.); lukas.krygier@regiomed-kliniken.de (L.K.); steffen.schnupp@regiomed-kliniken.de (S.S.); chrisitan.mahnkopf@regiomed-kliniken.de (C.M.); 2 Department of Cardiology and Angiology, Universitätsklinikum Essen, 45147 Essen, Germany; 3 Department of Cardiology, Istituto Cardiocentro Ticino, 6900 Lugano, Switzerland; amilzi@ukaachen.de; 4 Department of Ototrhinolaryngolgoy, Uniklinikum Mannheim, 68167 Mannheim, Germany; rakansaadoun@gmail.com

**Keywords:** sex, tricuspid valve regurgitation, transcatheter tricuspid valve repair, transcatheter edge-to-edge repair

## Abstract

Introduction: Tricuspid valve regurgitation (TR) is more prevalent among females. Transcatheter tricuspid valve repair (TTVR) using the edge-to-edge technique represents an alternative to surgery in patients with severe TR and high surgical risk. This study aims to investigate sex differences among patients undergoing TTVR. Methods: All patients who underwent TTVR at our center were retrospectively included. We compared baseline characteristics, intra-hospital, and one-year outcomes between males and females. Results: A total of 105 consecutive patients underwent TTVR. Females were more prevalent in the study cohort (n = 63, 60%). Coronary artery disease (CAD) was more evident in males than females (71.4% vs. 47.6%, *p* = 0.016). Left ventricular ejection fraction (LVEF) was also worse in males (48.8 ± 13.4 vs. 58 ± 6.8, *p* < 0.001). Other clinical characteristics were similar between both groups. The Success of the procedure (88.1% vs. 95.2%, *p* = 0.177) and intra-hospital mortality (4.8% vs. 11.1%, *p* = 0.255) were similar among males and females. At one-year follow-up, mortality was similar between both groups (24.3% vs. 25.9%, *p* = 0.863). Furthermore, hospitalization due to acute heart failure was also similar between both groups (40.5% vs. 37.5%, *p* = 0.768), as was a composite endpoint of death or hospitalization. In patients with successful procedures and who survived one year, TR severity was comparable between both groups. Conclusions: In our real-world cohort, more females underwent TTVR than males. No difference was observed in outcomes between males and females at one-year follow-up.

## 1. Introduction

Sex differences between males and females among patients with valvular heart disease are well known and include differences in epidemiology, pathophysiology, and outcomes [1]. In epidemiological studies, tricuspid valve regurgitation (TR) was more prevalent in females [2,3,4]. Furthermore, in patients undergoing valvular cardiac surgery, female sex was associated with increased intra-hospital and long-term mortality [5,6]. Transcatheter treatment has emerged as an alternative to surgery in patients with significant TR and high surgical risk. Among different treatment options, tricuspid valve repair using the edge-to-edge technique (TEER) represents the most applied treatment [7,8]. In a previous study comparing outcomes between males and females undergoing any type of transcatheter tricuspid valve intervention, mid-term mortality was similar [9]. To our knowledge, there are no data comparing outcomes between males and females in patients undergoing tricuspid TEER. This retrospective observational study aims to assess sex differences among patients undergoing tricuspid valve TEER.

## 2. Methods

Patient selection: All patients who underwent tricuspid valve TEER at our center between November 2020 and March 2023 were retrospectively included. All patients suffered from symptomatic severe TR as defined and classified by current guidelines [10,11,12]. The severity of TR was assessed using transthoracic and transesophageal echocardiography. All patients were evaluated by the heart team and deemed non-operable. The heart team assessed the morphology of the tricuspid valve and the mechanism of regurgitation to determine eligibility for TEER [13,14]. In all cases, the multidisciplinary heart team recommended proceeding with tricuspid TEER.

Informed consent was obtained from each patient. The study protocol conforms to the ethical guidelines of the 1975 Declaration of Helsinki as reflected in prior approval by the institution’s human research committee.

Transcatheter tricuspid valve repair: Interventions were performed under general anesthesia and were guided by transesophageal echocardiography in addition to fluoroscopy. All procedures were performed using dedicated devices: TriClip^®^ (Abbott, IL, USA) or Pascal^®^ (Edwards Lifesciences, Irvine, CA, USA) devices. The transcatheter tricuspid valve TEER procedure has been previously described [15].

Definition of outcomes and Follow-up: intra-hospital outcomes included the success of the procedure, defined as the successful implantation of at least one clip and a reduction of TR by at least one grade, vascular complications, and in hospital mortality.

**Figure 1:**
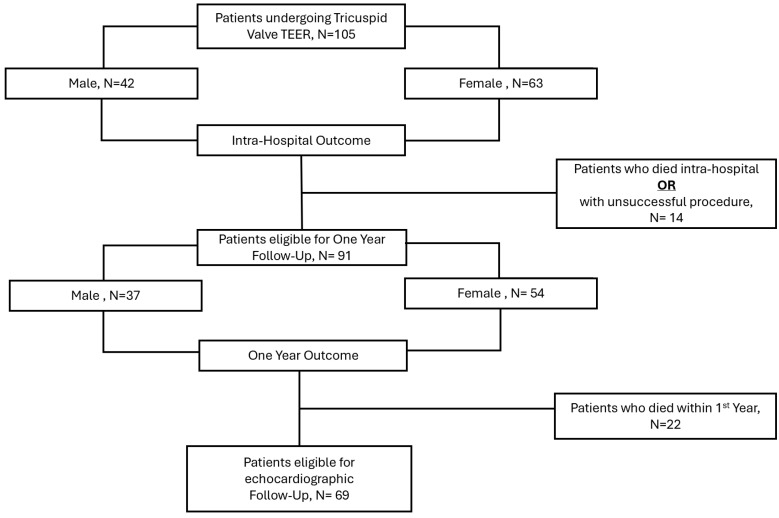
Flow chart of the study.

One-year outcomes included all-cause mortality, rehospitalization due to acute decompensated heart failure, and a composite endpoint of death or rehospitalization. In patients with a successful procedure and who survived at the one-year follow-up, the severity of TR was assessed. A flowchart of the study is supplied in Figure 1.

Follow-up data were obtained retrospectively from patients’ medical records. Patients who did not have follow-up data in our data management system were contacted by telephone and asked about rehospitalization due to acute heart failure. In cases where patients could not be reached, the primary care physician or the patients’ family was contacted to gather information on mortality and rehospitalization. For patients who did not survive, the date of death was obtained through their primary care physician. For patients who did not have an echocardiographic follow-up at our center, the severity of TR was obtained from the treating cardiologist.

## 3. Statistical Analysis

Continuous variables were expressed as mean ± standard deviation, and binary variables were expressed as counts (percentages). Continuous variables were compared using the t-test for independent samples, and categorical variables were assessed by Pearson’s χ^2^ test.

To assess the association of sex with clinical outcomes (composite outcome of all-cause death or HF hospitalization, all-cause death, HF hospitalization), we performed a Cox logistic regression and plotted the results for female and male patients. Statistical analyses were performed with SPSS version 26.0 (IBM Corp., Armonk, NY, USA). Statistical significance was defined as *p* < 0.05.

## 4. Results

### 4.1. Patients’ Characteristics

One hundred and five consecutive patients (males = 42, 40%) were included. Age did not differ between males and females (80.7 ± 7.4 vs. 80.3 ± 5.8, *p* = 0.662). Coronary artery disease (CAD) was more evident in males than in females (71.4% vs. 47.6%, *p* = 0.016). Furthermore, males had a worse left ventricular ejection fraction (48.8 ± 13.4 vs. 58 ± 6.8, *p*< 0.001). RV dimensions were shown to be slightly larger in males than in females. Other baseline clinical characteristics, including diabetes mellitus, atrial fibrillation, arterial hypertension, renal insufficiency, the presence of a cardiac implantable electrical device, and previous valve interventions, were comparable between both groups. The severity of TR was also comparable between both groups. All baseline clinical and echocardiographic characteristics are summarized in Table 1.

### 4.2. Intra-Hospital Outcome

The procedure was successful in 97 patients (92.5%). The success of the procedure was similar between males and females (88.1% vs. 95.2%, *p* = 0.177). The number of implanted clips, the position of the clips, and the mean transvalvular pressure gradient were comparable between both groups. The presence of vascular complications as well as intra-hospital mortality did not differ between males and females (4.8% vs. 11.1%, *p* = 0.255). The postprocedural severity of TR was also comparable between both groups. No surgical conversion was needed in any patients.

**Figure 2:**
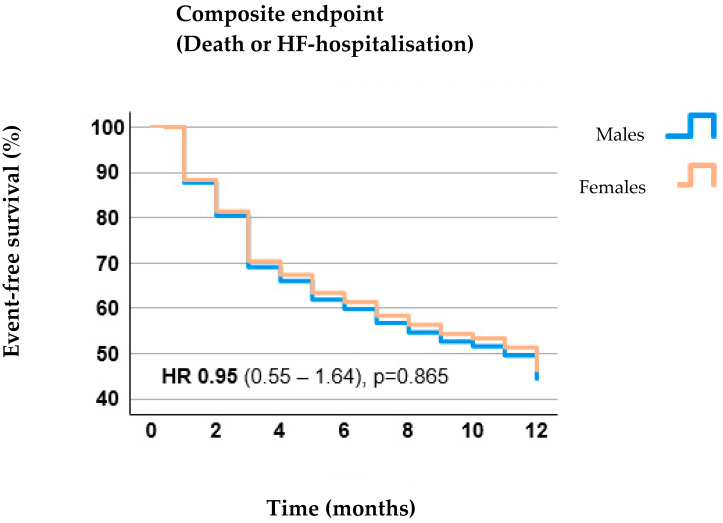

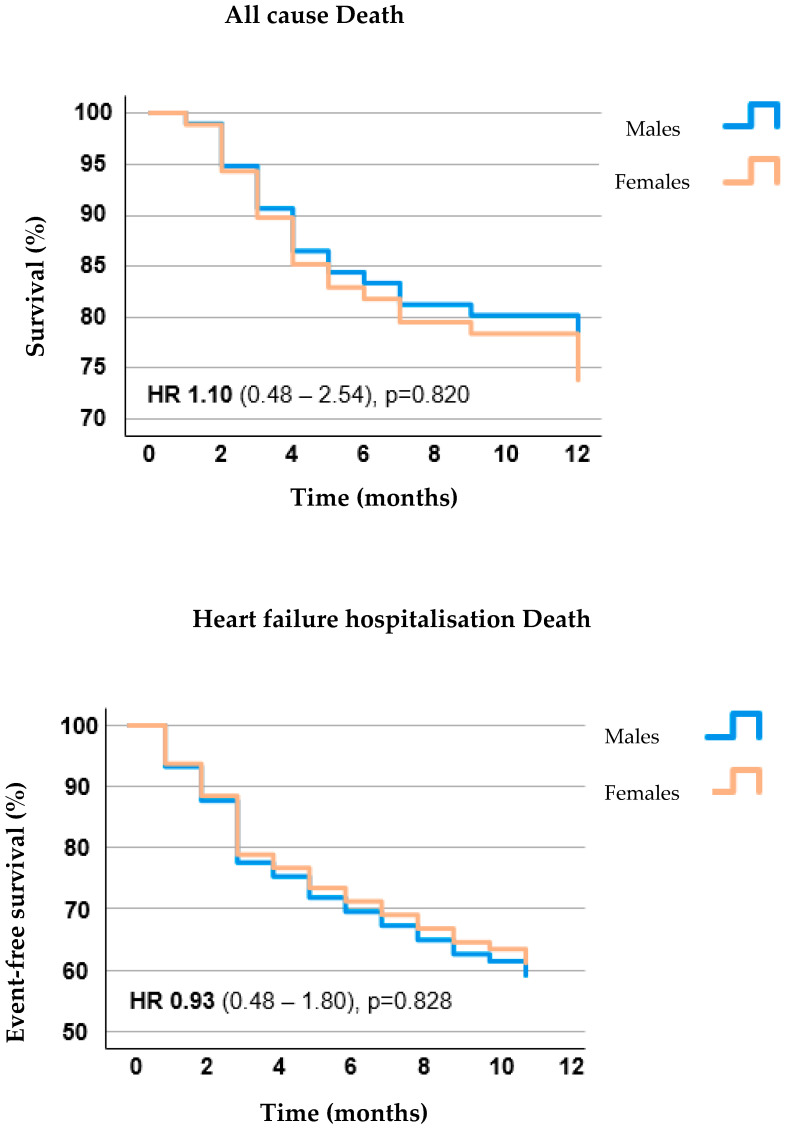
Comparison of one-year outcome between males und females.

**Figure 3:**
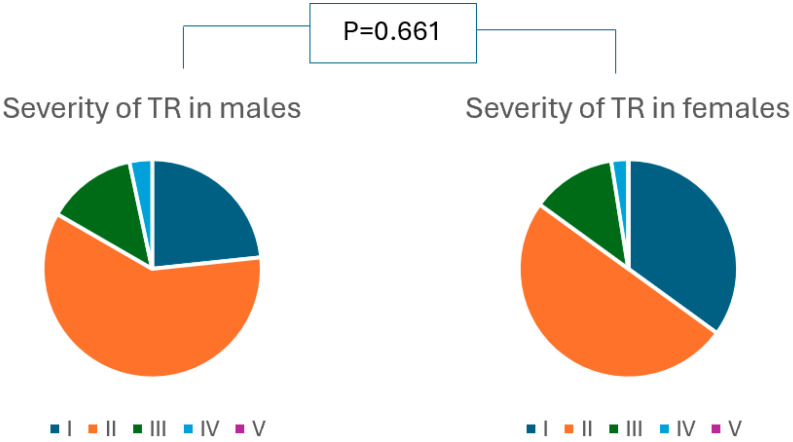
Comparison severity of TR at 1-year follow-up between males and females ^#^. ^#^ Included patients who had a successful procedure and who survived at one year.

One-year outcome: Mortality at one-year follow-up and hospitalization due to decompensated heart failure, as well as a composite endpoint of death or hospitalization due to decompensated heart failure, were comparable between males and females (Table 2, Figure 2). In patients who survived to the one-year follow-up, TR severity was comparable between both groups (Figure 3). No percutaneous or surgical re-intervention on the tricuspid valve was needed in any patient.

## 5. Discussion

The findings of our study suggest that female sex is more prevalent in patients undergoing tricuspid valve TEER. In epidemiological studies, TR was described as being more prevalent in females than in males, especially in the elderly. In the Framingham Heart Study, moderate to severe TR was prevalent in 5.6% of females aged more than 70 years, compared to 1.5% among males [2]. Toposky et al. showed that moderate TR is more prevalent in females aged more than 75 years compared to males [3]. Furthermore, Pfanmueller and colleagues stated that females represented 60% of patients undergoing tricuspid valve surgery [16]. In the dedicated RCTs investigating tricuspid valve TEER (TRILUMINATE and TRILUMINATE pivotal), females were also more represented than males [17,18]. In the international TriValve registry, females were more prevalent than males [7,9]. In the above-mentioned registry, the presence of CAD was more prevalent in males. In line with this finding, males in our cohort had a higher prevalence of CAD. In the study by Dietz et al., which aimed to assess differences in etiology, comorbidities, echocardiographic parameters, and prognosis between males and females with significant tricuspid regurgitation (TR), males had a worse LVEF, which was also in line with our findings [4]. Nevertheless, and contrary to our results, Dietz et al. found that females with relevant TR had a better prognosis regarding mortality and heart failure hospitalization in the short and long term compared to males. However, in this study, only 12–14% of the cohort underwent surgical therapy. Moreover, this prognostic benefit was eliminated after the propensity match between males and females. In our population, right ventricle dimensions were a little larger in males. However, this has been previously described in healthy volunteers [19]. Unfortunately, we did not have 3D-or CMR-derived measurements of RVDimensions or function, so we do not believe this difference in RV dimensions contributed to the outcomes. Although female sex is a risk factor in the EuroSCORE II [20], no sex-specific differences in mortality after TR surgery were found in the meta-analysis performed by Islam Khan et al. [21]. In patients undergoing any type of transcatheter tricuspid valve intervention, the success of the procedure as well as 2-year mortality did not differ between males and females [9]. In the PASTE registry, which reported the outcomes of patients undergoing tricuspid valve TEER with the Pascal^®^ (Edwards Lifesciences) device, no difference in the success of the procedure or survival was reported between males and females [22]. Similarly, our data suggest that tricuspid valve TEER was effective in both groups, as the success of the procedure, intra-hospital mortality, and one-year mortality were comparable. However, hospitalization due to decompensated acute heart failure was not reported in the study by Fortmeier et al. [9]. Our data did not show any difference between males and females regarding one-year mortality, hospitalization due to decompensated heart failure, or a composite of both endpoints. An ad hoc analysis of the dedicated RCTs may be of interest. However, one-year mortality after TTVR remains high. In our cohort, about one-quarter of patients died within one year. However, this result was comparable to other registries. In a meta-analysis performed by Sannino and colleagues, one-year mortality was also high and similar to our results [23]. It is worth to say, that about 40% of patients had more than severe TR. This high one-year mortality should warrant an earlier identification and treatment of TR.

Conclusively, our data suggest that tricuspid valve TEER is effective in both males and females, and the outcomes of the procedure do not differ between males and females.

## 6. Limitations

Although our study contributes significantly to understanding sex differences among patients undergoing tricuspid valve TEER in a real-world cohort, we acknowledge that it has limitations. The retrospective observational nature of the study represents the main limitation. Furthermore, as in any registry study, selection bias cannot be excluded. Most patients had secondary TR, and only a few patients had other types of TR [12]. Our study concentrated on hard endpoints, such as mortality and rehospitalization, but no data on functional outcomes or changes in quality of life were recorded. Lastly, this investigation suffers from the usual shortcomings of a single-center study.

## 7. Conclusions

In our real-world cohort, more females underwent TTVR than males. No difference in outcomes was observed between the groups at the one-year follow-up.

## Figures and Tables

**Table 1:** Clinical characteristics of the study population.

	All (n = 105)	Male (n = 42)	Female (n = 63)	*p*-Value
Age, years	80.0 ± 6.4	80.7 ± 7.4	80.3 ± 5.8	0.622
DM, n (%)	29 (27.6%)	9 (21.4%)	20 (31.7%)	0.247
HTN, n (%)	87 (82.9%)	35 (83.3%)	52 (82.5%)	0.916
Atrial fibrillation, n (%)	93 (88.6%)	37 (88.1%)	56 (88.9%)	0.900
Chronic renal failure, n (%)	82 (78.1%)	31 (73.8%)	51 (81.0%)	0.386
Dialysis, n (%)	4 (3.8%)	2 (4.8%)	2 (3.2%)	0.677
PAD	9 (8.6%)	4 (9.5%)	5 (7.9%)	0.776
COPD	17 (16.2%)	5 (11.9%)	12 (19.0%)	0.330
CAD, n (%)	60 (57.1%)	30 (71.4%)	30 (47.6%)	0.016
Previous cardiac surgery	10 (9.8%)	3 (7.1%)	7 (11.1%)	0.497
Previous mitral valve TEER, n (%)	25 (23.8%)	13 (31.0%)	12 (19.0%)	0.161
Previous TAVI	3 (2.9%)	1 (2.4%)	2 (3.2%)	0.811
CIED	25 (23.8%)	14 (33.3%)	11 (17.5%)	0.061
LVEF, %	54.0 ± 11.0	48.8 ± 13.4	57.8 ± 6.8	<0.001
TAPSE, mm	20.7 ± 5.1	20.0 ± 4.9	21.2 ± 4.9	0.260
RVEDD, mm	45.8 ± 5.5	47.2 ± 5.9	44.7 ± 4.9	0.048
TR severity				0.913
I	-	-	-	
II	-	-	-	
III	60 (57.1%)	24 (57.1%)	36 (57.1%)	
IV	41 (39.0%)	16 (38.1%)	25 (39.7%)	
V	4 (3.8%)	2 (4.8%)	2 (3.2%)	
Machanism of TR				0.795
Primary	4 (3.8%)	1 (2.3)	3 (4.8)	
Secondary	98 (93.4)	40 (95.4)	58 (92)	
CIED induced	3 (2.8)	1 (2.3)	2 (3.2)	
Used device TriClip^®^ (Abbott)	101 (95.2)	40 (95.2)	61 (96.8)	1

DM: diabetes mellitus, HTN: arterial hypertension, PAD: peripheral artery disease, COPD: chronic obstructive pulmonary disease, CAD: coronary artery disease, TEER: transcatheter edge-to-edge repair, TAVI: transcatheter aortic valve implantation, CIED: cardiac implanted electrical devices, LVEF: left ventricular ejection fraction, TAPSE: tricuspid annular plane systolic excursion, RVEDD: right ventricle end diastolic diameter, TR: tricuspid regurgitation.

**Table 2:** Intra-hospital and one-year outcome.

	Intra-Hospital Outcome (n = 105)			
	All (n = 105)	Male (n = 42)	Female (n = 63)	*p*-Value
Success of the procedure	97 (92.4%)	37 (88.1%)	60 (95.2%)	0.177
Number of clips, n (%)				0.151
1	56 (53.3)	38 (60.3)	18 (42.9)	
2	40 (38.1)	20 (47.6)	20 (31.7)	
3	2 (1.9)	0	2 (3.2)	
Position of clip				0.794
Antero-septal	68 (64.8)	27 (64.3)	41 (65.1)	
Postero-septal	17 (16.2)	6 (14.3)	11 (17.5)	
Both	13 (12.4)	5 (11.9)	8 (12.7)	
Pmean, mmHG	2.8 ± 1.3	2.7 ± 1.2	2.9 ± 1.3	0.506
Vascular complications, n (%)	10 (9.5)	2 (4.8)	8 (12.7)	0.175
Stroke	0(0)	0(0)	(0)	-
Intra-hospital mortality, n (%)	9 (8.6)	2 (4.8)	7 (11.1)	0.255
TR severity				0.168
I	51 (48.6)	23 (54.8)	28 (44.4)	
II	43 (41)	14 (33.3)	29 (46.0)	
III	6 (5.7)	1(2.4)	5 (7.9)	
IV	4 (3.8)	3 (7.1)	1 (1.6)	
V	1 (1)	1 (2.4)	0 (0)	
	**One-Year Outcome (n = 91) ^+^**			
	**All** **(n = 91)**	**Male** **(n = 37)**	**Female** **(n = 54)**	***p*-Value**
Mortality at one year (n,%)	23 (25.3)	9 (24.3)	14 (25.9)	0.863
HF hospitalization (n, %)	36 (38.7)	15 (40.5)	21 (37.5)	0.768

**^+^** Included patients who had a successful procedure and who survived intra-hospital. TR: tricuspid regurgitation.

## Data Availability

The raw data supporting the conclusions of this article will be made available by the authors on request.
